# The value of thromboelastography in evaluating the efficacy of Xueshuantong combined with edaravone in the treatment of acute cerebral infarction

**DOI:** 10.1097/MD.0000000000037954

**Published:** 2024-04-26

**Authors:** Yu Wang, Litao Li, Xiaojie Hu, Liqiu Huang, Zheng Li

**Affiliations:** a Department of Laboratory, Tangshan Central Hospital, Tangshan City, Hebei Province, China; b Internal Medicine Department 1, Zhao County People’s Hospital, Shijiazhuang City, Hebei Province, China; c Department of Emergency, Affiliated Hospital of North China University of Science and Technology, Tangshan City, Hebei Province, China.

**Keywords:** acute cerebral infarction, edaravone, thromboelastography, Xueshuantong

## Abstract

To explore the value of thromboelastography (TEG) in evaluating the efficacy of Xueshuantong combined with edaravone for the treatment of acute cerebral infarction (ACI). We retrospectively analyzed the clinical data of 96 patients with ACI treated with Xueshuantong combined with edaravone and monitored by TEG. The correlation between the results of TEG examination and treatment outcomes in patients after treatment was analyzed. After treatment, 65 of 96 patients showed good efficacy and 31 had poor efficacy. kinetic time (KT), reaction time (RT), and the percentage of clot lysis at 30 minutes after Ma value (LY30) of patients with good therapeutic effects were significantly higher than those with poor therapeutic effects; However, maximum amplitude (MA) and coagulation index (CI) were significantly lower than those with poor efficacy (*P* < .05). There was a significant positive correlation between KT, RT, and LY30 and the therapeutic effect of ACI, and a significant negative correlation between the therapeutic effects of MA, CI, and ACI (*P* < .05). Logistic analysis confirmed that KT, RT, and LY30 were protective factors for the therapeutic effect of ACI; MA and CI were risk factors for the therapeutic effect of ACI (*P* < .05). TEG has a high value in evaluating the efficacy of Xueshuantong combined with edaravone in the treatment of ACI. It can clarify changes in the coagulation function of patients, thereby guiding clinical follow-up treatment.

## 1. Introduction

Acute cerebral infarction (ACI) is a common cerebrovascular disease that is more common in middle-aged and elderly populations.^[1]^ In recent years, its incidence rate continues to increase, and the number of patients is getting younger, which has become a serious threat to people’s physical and mental health and quality of life of social public health problems.^[1,2]^ Edaravone and Xueshuantong are both important therapeutic drugs for ACI.^[3–5]^ Among them, edaravone belongs to the category of neuroprotective agents and brain tissue free radical scavengers, capable of clearing oxygen free radicals and antioxidant properties, can clear hydroxyl groups in the brain after ischemia-reperfusion, inhibit delayed neuronal death in the infarcted area, and relieve adverse signs and symptoms of cerebral edema.^[3,4]^ Xueshuantong is a traditional Chinese medicine preparation that removes blood stasis, stops bleeding, promotes blood circulation, and removes blood stasis. It can alleviate ischemia-reperfusion injury, eliminate oxygen free radicals, improve plaque stability, antagonize atherosclerosis, and regulate hemodynamics.^[5,6]^

However, shock is prone to occur during ACI treatment, causing activation and disorder of the coagulation system and varying degrees of damage to vascular endothelial cells.^[7]^ Moreover, the massive release of clotting substances by the coagulation system can cause microcirculatory disorders in brain tissue, affecting disease outcomes.^[8,9]^ Therefore, it is of great significance to clarify coagulation function status during ACI treatment. Thromboelastography (TEG) is an important tool for evaluating the coagulation function. It dynamically reflects fibrinolysis, platelet function, and coagulation factor status throughout the coagulation process. Evaluating coagulation function through reaction time (RT) and maximum amplitude (MA) has high sensitivity.^[10,11]^

Based on this, this study retrospectively analyze the clinical data of patients who received Xueshuantong combined with edaravone and were monitored by TEG in our hospital. This study aimed to clarify the evaluation value of TEG in the treatment of ACI with Xueshuantong combined with edaravone.

## 2. Materials and methods

A retrospective analysis of 96 patients who received Xueshuantong combined with edaravone in our hospital from June 2021 to June 2023 was performed, and clinical outcomes were monitored using TEG. There were 59 males and 37 females, with age range from 42 to 71 years old and an average of 54.75 ± 6.10 years old.

*Inclusion criteria:*

- Patients met the diagnostic criteria of ACI confirmed by CT scan or MRI examination;^[1]^
- The interval from onset to admission was < 24 hours;
- TEG data was complete.

*Exclusion criteria:*

- Patients treated with thrombin inhibitors, sodium citrate, heparin, etc during prehospital rescue;
- Patients with organic lesions in kidney, liver and other organs;
- Those with hematological diseases;
- Those who received vascular intervention/ intravenous thrombolysis after admission;
- Patients with cerebral hemorrhage;
- Allergic constitution.

Therapeutic method: Edaravone (Sinopharm Guorui Pharmaceutical Co., Ltd., Sinopharm Zhunzi h20080056, specification: 20 mL: 30 mg) 30 mg + normal saline 100 mL was intravenously administered twice daily. Xueshuantong (Guangxi Wuzhou Pharmaceutical Group Co., Ltd., gyzz z20025652, specification: 150 mg) 250 mg + 250 mL normal saline once a day. The treatment was continued for 14 days.

TEG: T-400 thromboelastograph and supporting kit (Guangzhou Yangpu Medical Technology Co., Ltd.). Pre-elbow venous blood was collected on an empty stomach in the morning, placed in a sodium citrate anticoagulant tube, and the following indicators were detected within 2H. Take sodium citrate anticoagulant, mix well, draw 1000 tb sample from the sampling gun, add kaolin activation tube, mix well and stand for 4 minutes, take 340 btl liquid and 20 tacacl reagent to the ordinary cup; The following indicators were detected on the machine after shock: RT; kinetic time (KT); MA; percentage of clot lysis at 30 minutes after MA (LY30); coagulation index (CI). All operating steps were carried out strictly in accordance with the instructions of the instrument and reagent.

Evaluation of clinical effect: The clinical effect was evaluated according to the change of National institutes of health stroke scale (NIHSS) score. The NIHSS score decreased by 91% to 100%, and the degree of disability of 0 indicated basically recovery; The NIHSS score decreased by 46% to 90%, and the degree of disability of 1 to 3 indicated significantly improved; The NIHSS score decreased by 18% to 45% indicated progress; The NIHSS score decreased or increased > 17% indicated no change; The increase of NIHSS score > 18% indicated a deterioration. Basically recovery, significantly improved, and the effect of progress were classified as good effect, while no change and deterioration were classified as poor effect.^[12]^

Statistical methods: All data were analyzed using the SPSS software (version 25.0; IBM Corp., Armonk, NY, USA). The measurement data are expressed as the mean ± standard deviation, and the independent sample *t* test was used for comparison between groups. The counting data were expressed by the number of cases, and compared using Chi-square test. Spearman’s correlation was used to analyze the correlation between TEG and ACI treatment effect, and the risk factors of ACI treatment effect were analyzed by logistic regression. *P* < .05, indicating that the difference was statistically significant.

## 3. Results

A total of 96 patients met the inclusion criteria, with 65 cases of good effects and 31 cases of poor effects. There was no significant difference in the baseline data between the 2 groups (*P* > .05) (Table 1). KT, RT, and LY30 of patients with good effects were significantly higher than those with poor effects, while MA and CI were significantly lower than those with poor effects (*P* < .05) (Table 2). Spearman analysis showed that KT, RT, and LY30 were significantly positively correlated with the therapeutic effect of ACI, while MA and CI were significantly negatively correlated with the therapeutic effect of ACI (*P *< .05) (Table 3). The logistic model showed that KT, RT, and LY30 were protective factors for ACI treatment effect, While MA and CI were risk factors for ACI treatment effect (*P* < .05) (Table 4).

**Table 1:** Comparison of baseline data of patients with good and poor effects.

Baseline data	Poor effect (n = 31)	Good effect (n = 65)	*χ^2^/t*	*P*
Gender (male/female)	18/13	40/25	0.106	.745
Age (yr)	55.87 ± 6.26	54.22 ± 6.00	1.247	.216
Hypertension (yes)	14	25	0.391	.532
Diabetes (yes)	11	14	2.119	.145
Smoking (yes)	12	15	0.168	.682
Drinking (yes)	12	19	0.863	.353
NIHSS at admission (score)	15.68 ± 3.21	16.42 ± 3.00	−1.102	.273

**Table 2:** Comparison of TEG examination results of ACI patients with different clinical effects.

Clinical effect	n	KT (min)	RT (min)	LY30 (%)	MA (mm)	CI
Poor effect	7	1.24 ± 0.56	4.00 ± 0.82	4.65 ± 0.80	70.94 ± 8.13	2.16 ± 0.69
Good effect	53	2.28 ± 0.57	5.34 ± 1.08	5.28 ± 0.82	59.68 ± 8.38	1.35 ± 0.57
*χ^2^*		−8.392	−6.741	−3.561	6.213	6.058
*P*		<.001	<.001	.001	<.001	<.001

*Note:* ACI = acute cerebral infarction, coagulation time, CI = coagulation index, KT = coagulation time, LY30 = the percentage of clot lysis at 30 minutes after Ma value, MA = maximum amplitude, RT = reaction time, TEG = thromboelastography.

**Table 3:** Correlation analysis between TEG index and treatment effect.

Item		KT	RT	LY30	MA	CI
Treatment effect	*r*	0.651	0.541	0.332	−0.520	−0.531
	*P*	<.001	<.001	.001	<.001	<.001

*Note:* CI = coagulation index, KT = coagulation time, LY30 = the percentage of clot lysis at 30 minutes after Ma, MA = maximum amplitude, RT = reaction time, TEG = thromboelastographyvalue.

**Table 4:** Logistic analysis of influencing factors of ACI treatment effect.

Variable	B	S.E.	Wald *χ^2^*	*P*	OR	95% CI
KT	2.371	0.989	5.751	.016	10.707	1.542–74.337
RT	1.463	0.685	4.554	.033	4.318	1.127–16.548
LY30	2.005	0.92	4.752	.029	7.423	1.224–45.015
MA	−0.218	0.078	7.811	.005	0.804	0.690–0.937
CI	−1.977	0.874	5.114	.024	0.138	0.025–0.768

*Note:* ACI = acute cerebral infarction, CI = coagulation index, KT = coagulation time, LY30 = the percentage of clot lysis at 30 minutes after Ma value, MA = maximum amplitude, RT = reaction time.

## 4. Discussion

The results of this study showed that ACI treatment could be determined using TEG. It is helpful to guide the clinical formulation or adjustment of follow-up intervention programs, ensure that patients receive targeted treatment, and thus, promote a good prognosis of the disease. Yuan et al^[13]^ and others conducted a comparative analysis of the relevant indicators of TEG in ACI patients and healthy people; The results showed that compared with the healthy control group, the CI value and MA value of ACI patients were increased, while KT and RT were significantly decreased. Therefore, TEG parameters are considered sensitive indicators of the hypercoagulable state in patients with ACI. It can be used to evaluate a patient’s condition, coagulation function, and treatment effects.^[13,14]^ It is consistent with the conclusions of this study. Rowe et al^[15]^ performed a comparative analysis of TEG in patients with acute ischemic stroke after alteplase treatment; 7 patients were included in the study, and the related indicators of TEG were in the normal range at baseline. Fibrin accumulation was significantly inhibited 30 minutes after starting alteplase infusion, and the lowest MA value was also abnormal 60 minutes after alteplase infusion. After 150 minutes of alteplase infusion, the levels of TEG-related indicators returned to the baseline level. Therefore, it can be confirmed that TEG can clarify the changes of coagulation system in patients with acute ischemic stroke after alteplase treatment. We also predicted whether patients would have a higher risk of adverse events.^[15,16]^ Shimei et al^[17]^ showed that the levels of TEG-related indicators in ACI patients were higher or lower than those in healthy controls, and with the severity of the disease. Logistic regression analysis confirmed that the relevant indicators of TEG were important risk factors affecting the degree of brain injury in patients with cerebral infarction. The study also pointed out that TEG can effectively and accurately evaluate the platelet aggregation rate of ACI patients and timely determine the reactivity of antiplatelet drugs in patients with cerebral infarction; to guide the clinical development of individualized antiplatelet therapy, timely use of highly responsive antithrombotic drugs to replace easily resistant drugs, and to improve the therapeutic effect of antithrombotic therapy in patients, it can reduce the drug dose to a certain extent, improve the fibrinolysis and coagulation function of the body, and minimize the high bleeding risk of combined drugs.^[16,17]^ Jin et al^[18]^ also pointed out that personalized treatment plans based on TEG monitoring can guide the clinical use of highly responsive antiplatelet drugs, regulate blood microcirculation in the ischemic area of the brain, inhibit cerebral thrombosis, reduce inflammation, improve endothelial function, and improve quality of life. In addition, TEG can dynamically monitor the changes in blood viscosity during the coagulation process of patients and analyze the entire coagulation fibrinolysis process in graphical form, providing more comprehensive and intuitive indicators, helping physicians comprehensively grasp coagulation function and monitor the patient’s disease treatment status.^[19,20]^

This study’s logistic analysis confirmed that KT, RT, and LY30 were protective factors for ACI treatment efficacy, while MA and CI were risk factors for ACI treatment efficacy. Clinical practice can refer to the monitoring of TEG-related indicators in ACI patients for corresponding treatment, improving the coagulation function status of patients, and ensuring the treatment and prognosis of the disease.^[21,22]^

Limitations: First, this was a single-center, retrospective study. Incomplete medical records and bias in recalling medical history increase the complexity of the research and may lead to selection bias. Second, the small sample size and limited observation indicators are also shortcomings of this retrospective study. Third, the prognosis of patients was not analyzed. Fourth, the role of TEG in the diagnosis and treatment of mild to moderate acute cerebral infarction needs to be confirmed through large-scale and more rigorous controlled studies.

## 5. Conclusion

TEG has a high value in evaluating the efficacy of Xueshuantong combined with edaravone in the treatment of ACI. It can clarify changes in the coagulation function of patients, thereby guiding clinical follow-up treatment.

## Author contributions

**Conceptualization:** Yu Wang.

**Data curation:** Litao Li.

**Formal analysis:** Xiaojie Hu.

**Investigation:** Liqiu Huang, Zheng Li.

**Writing – original draft:** Yu Wang.

**Writing – review & editing:** Yu Wang.
